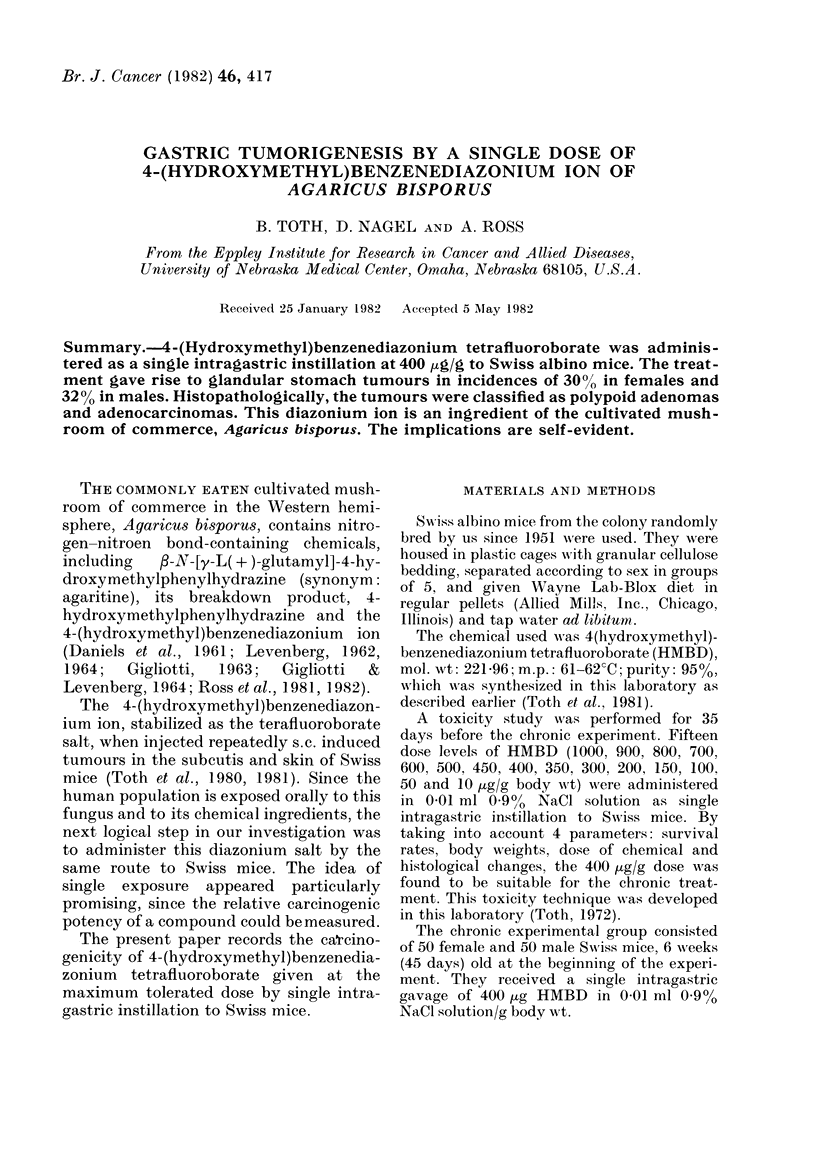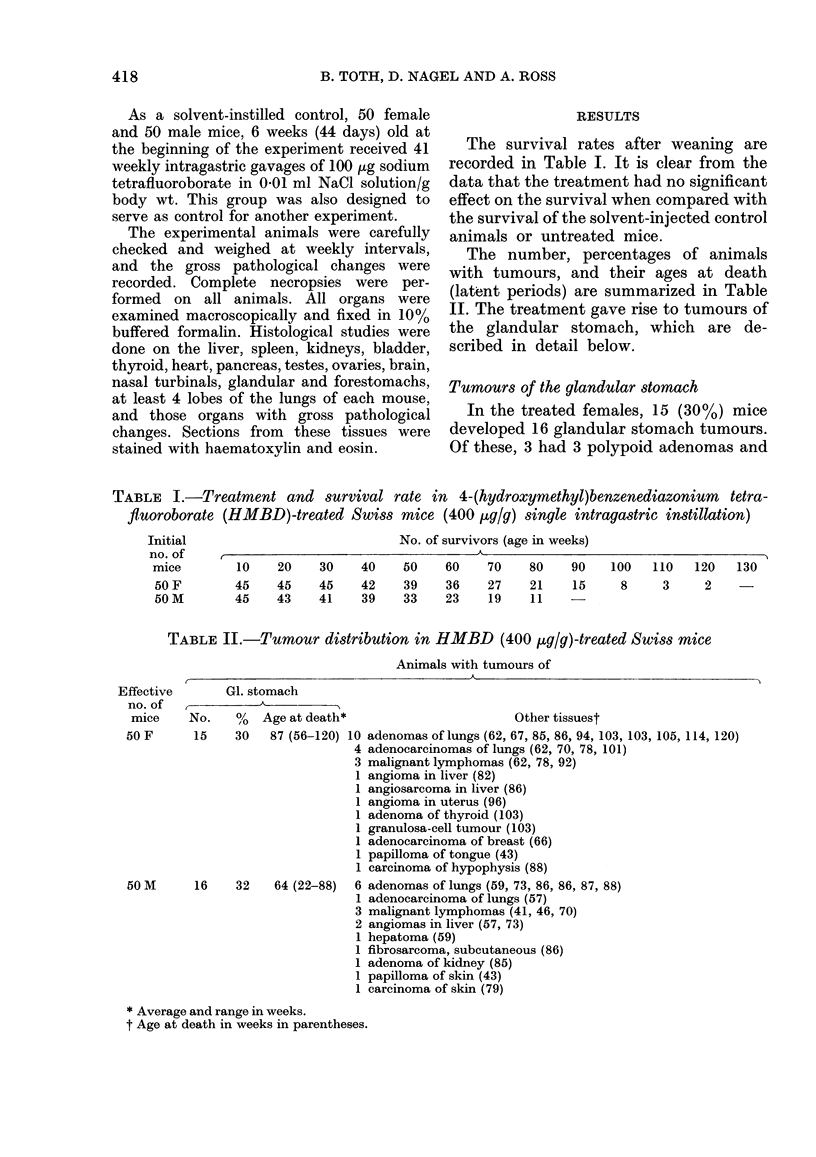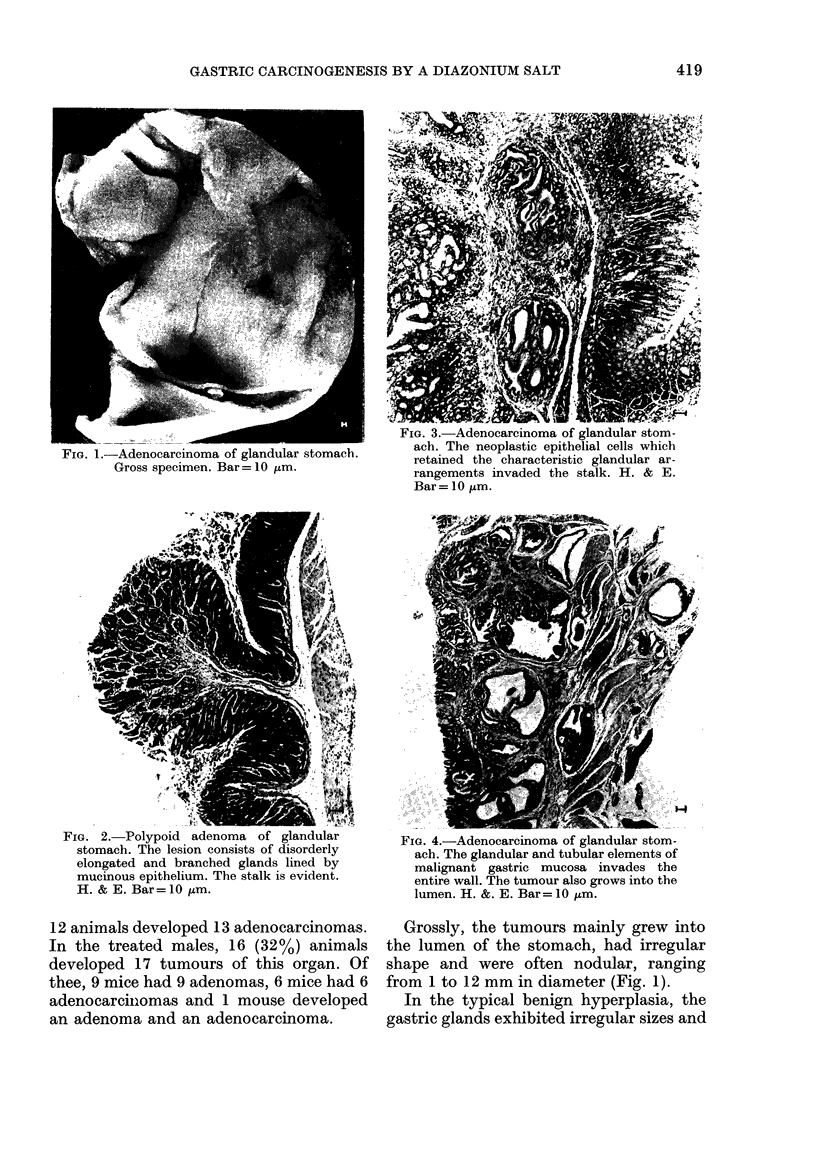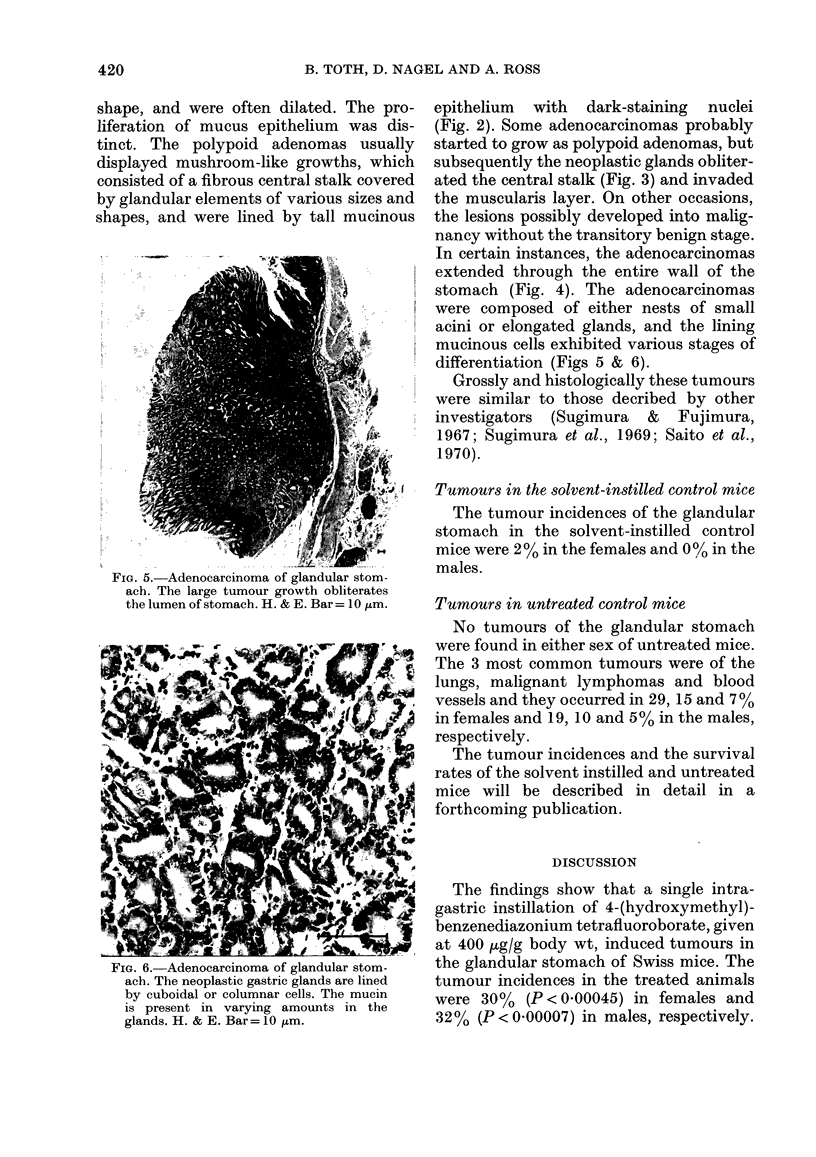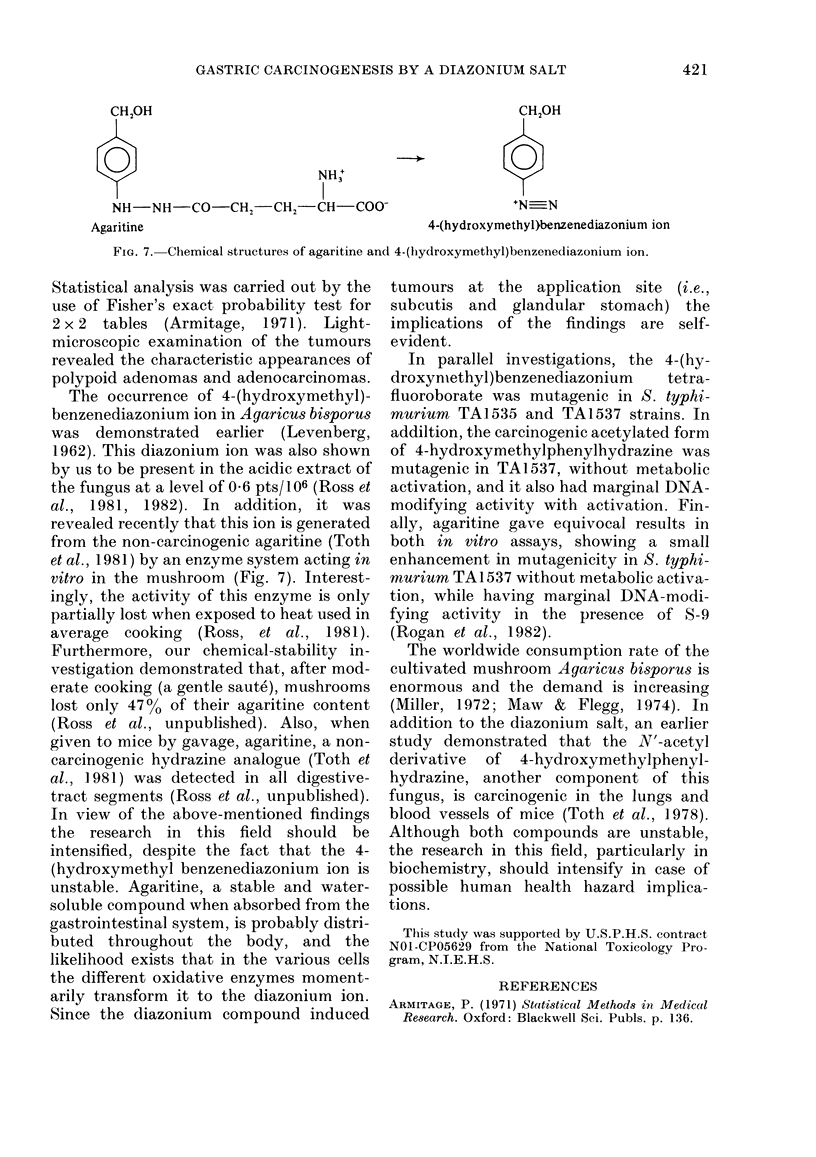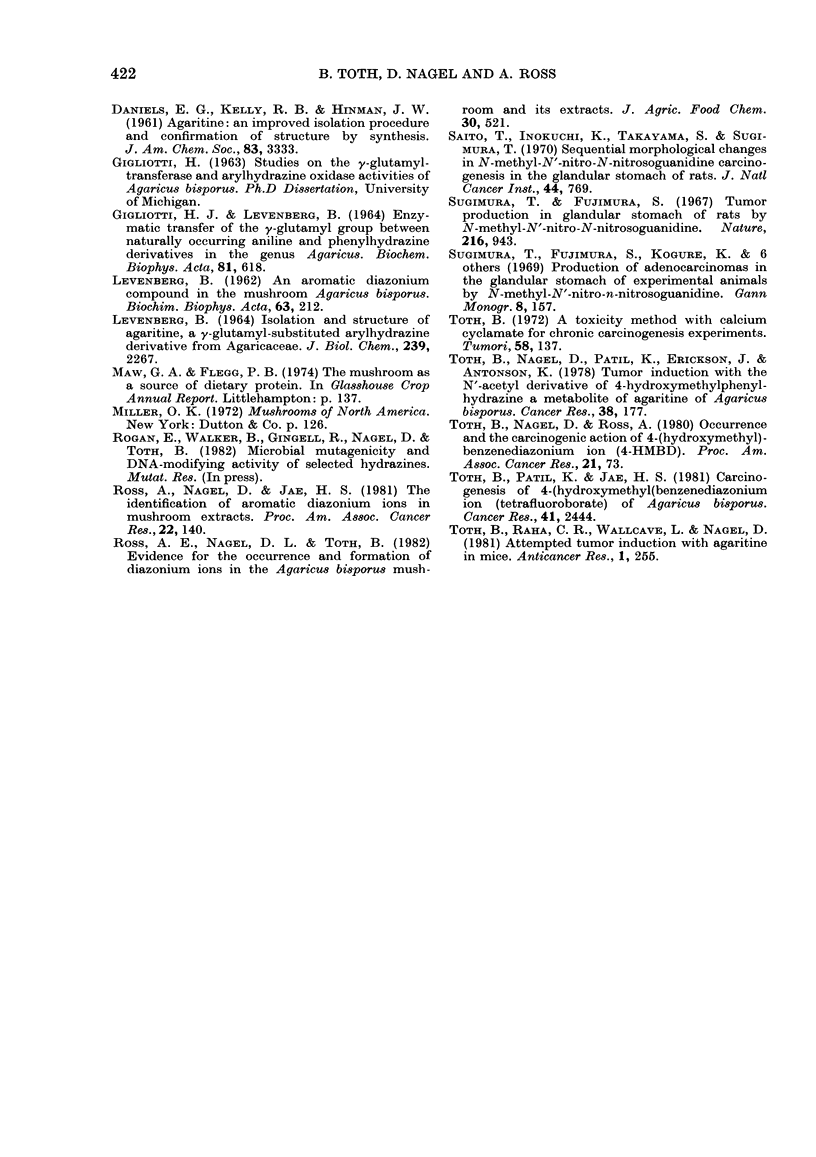# Gastric tumorigenesis by a single dose of 4-(hydroxymethyl)benzenediazonium ion of Agaricus bisporus.

**DOI:** 10.1038/bjc.1982.218

**Published:** 1982-09

**Authors:** B. Toth, D. Nagel, A. Ross

## Abstract

**Images:**


*Br. J. Cancer* (1982) **46**, 417

### GASTRIC TUMORIGENESIS BY A SINGLE DOSE OF 4-(HYDROXYMETHYL)BENZENEDIAZONIUM ION OF *AGARICUS BISPORUS*

B. TOTH, D. NAGEL AND A. ROSS

*From the Eppley Institute for Research in Cancer and Allied Diseases, University of Nebraska Medical Center, Omaha, Nebraska* 68105, *U.S.A.*

Received 25 January 1982 Accepted 5 May 1982

**Summary.—4-(Hydroxymethyl)benzenediazonium tetrafluoroborate was administered as a single intragastric instillation at 400 μg/g to Swiss albino mice. The treatment gave rise to glandular stomach tumours in incidences of 30% in females and 32% in males. Histopathologically, the tumours were classified as polypoid adenomas and adenocarcinomas. This diazonium ion is an ingredient of the cultivated mushroom of commerce, *Agaricus bisporus*. The implications are self-evident.**

THE COMMONLY EATEN cultivated mushroom of commerce in the Western hemisphere, *Agaricus bisporus*, contains nitrogen–nitroen bond-containing chemicals, including β-*N*-[γ-L(+)-glutamyl]-4-hydroxymethylphenylhydrazine (synonym: agaritine), its breakdown product, 4-hydroxymethylphenylhydrazine and the 4-(hydroxymethyl)benzenediazonium ion (Daniels *et al.*, 1961; Levenberg, 1962, 1964; Gigliotti, 1963; Gigliotti & Levenberg, 1964; Ross *et al.*, 1981, 1982).

The 4-(hydroxymethyl)benzenediazonium ion, stabilized as the terafluoroborate salt, when injected repeatedly s.c. induced tumours in the subcutis and skin of Swiss mice (Toth *et al.*, 1980, 1981). Since the human population is exposed orally to this fungus and to its chemical ingredients, the next logical step in our investigation was to administer this diazonium salt by the same route to Swiss mice. The idea of single exposure appeared particularly promising, since the relative carcinogenic potency of a compound could be measured.

The present paper records the carcinogenicity of 4-(hydroxymethyl)benzenediazonium tetrafluoroborate given at the maximum tolerated dose by single intragastric instillation to Swiss mice.

### MATERIALS AND METHODS

Swiss albino mice from the colony randomly bred by us since 1951 were used. They were housed in plastic cages with granular cellulose bedding, separated according to sex in groups of 5, and given Wayne Lab-Blox diet in regular pellets (Allied Mills, Inc., Chicago, Illinois) and tap water *ad libitum*.

The chemical used was 4(hydroxymethyl)-benzenediazonium tetrafluoroborate (HMBD), mol. wt: 221·96; m.p.: 61–62°C; purity: 95%, which was synthesized in this laboratory as described earlier (Toth *et al.*, 1981).

A toxicity study was performed for 35 days before the chronic experiment. Fifteen dose levels of HMBD (1000, 900, 800, 700, 600, 500, 450, 400, 350, 300, 200, 150, 100, 50 and 10 μg/g body wt) were administered in 0·01 ml 0·9% NaCl solution as single intragastric instillation to Swiss mice. By taking into account 4 parameters: survival rates, body weights, dose of chemical and histological changes, the 400 μg/g dose was found to be suitable for the chronic treatment. This toxicity technique was developed in this laboratory (Toth, 1972).

The chronic experimental group consisted of 50 female and 50 male Swiss mice, 6 weeks (45 days) old at the beginning of the experiment. They received a single intragastric gavage of 400 μg HMBD in 0·01 ml 0·9% NaCl solution/g body wt.

As a solvent-instilled control, 50 female and 50 male mice, 6 weeks (44 days) old at the beginning of the experiment received 41 weekly intragastric gavages of 100 μg sodium tetrafluoroborate in 0·01 ml NaCl solution/g body wt. This group was also designed to serve as control for another experiment.

The experimental animals were carefully checked and weighed at weekly intervals, and the gross pathological changes were recorded. Complete necropsies were performed on all animals. All organs were examined macroscopically and fixed in 10% buffered formalin. Histological studies were done on the liver, spleen, kidneys, bladder, thyroid, heart, pancreas, testes, ovaries, brain, nasal turbinals, glandular and forestomachs, at least 4 lobes of the lungs of each mouse, and those organs with gross pathological changes. Sections from these tissues were stained with haematoxylin and eosin.

### RESULTS

The survival rates after weaning are recorded in Table I. It is clear from the data that the treatment had no significant effect on the survival when compared with the survival of the solvent-injected control animals or untreated mice.

The number, percentages of animals with tumours, and their ages at death (latent periods) are summarized in Table II. The treatment gave rise to tumours of the glandular stomach, which are described in detail below.

### *Tumours of the glandular stomach*

In the treated females, 15 (30%) mice developed 16 glandular stomach tumours. Of these, 3 had 3 polypoid adenomas and

TABLE I.—*Treatment and survival rate in 4-(hydroxymethyl)benzenediazonium tetrafluoroborate (HMBD)-treated Swiss mice (400 μg/g) single intragastric instillation)*

| Initial no. of mice | No. of survivors (age in weeks) | | | | | | | | | | | | |
|---|---|---|---|---|---|---|---|---|---|---|---|---|---|
| | 10 | 20 | 30 | 40 | 50 | 60 | 70 | 80 | 90 | 100 | 110 | 120 | 130 |
| 50 F | 45 | 45 | 45 | 42 | 39 | 36 | 27 | 21 | 15 | 8 | 3 | 2 | — |
| 50 M | 45 | 43 | 41 | 39 | 33 | 23 | 19 | 11 | — | | | | |

TABLE II.—*Tumour distribution in HMBD (400 μg/g)-treated Swiss mice*

| Effective no. of mice | Animals with tumours of: Gl. stomach No. | % | Age at death* | Other tissues† |
|---|---|---|---|---|
| 50 F | 15 | 30 | 87 (56–120) | 10 adenomas of lungs (62, 67, 85, 86, 94, 103, 103, 105, 114, 120) |
| | | | | 4 adenocarcinomas of lungs (62, 70, 78, 101) |
| | | | | 3 malignant lymphomas (62, 78, 92) |
| | | | | 1 angioma in liver (82) |
| | | | | 1 angiosarcoma in liver (86) |
| | | | | 1 angioma in uterus (96) |
| | | | | 1 adenoma of thyroid (103) |
| | | | | 1 granulosa-cell tumour (103) |
| | | | | 1 adenocarcinoma of breast (66) |
| | | | | 1 papilloma of tongue (43) |
| | | | | 1 carcinoma of hypophysis (88) |
| 50 M | 16 | 32 | 64 (22–88) | 6 adenomas of lungs (59, 73, 86, 86, 87, 88) |
| | | | | 1 adenocarcinoma of lungs (57) |
| | | | | 3 malignant lymphomas (41, 46, 70) |
| | | | | 2 angiomas in liver (57, 73) |
| | | | | 1 hepatoma (59) |
| | | | | 1 fibrosarcoma, subcutaneous (86) |
| | | | | 1 adenoma of kidney (85) |
| | | | | 1 papilloma of skin (43) |
| | | | | 1 carcinoma of skin (79) |

* Average and range in weeks.
† Age at death in weeks in parentheses.

Fig. 1.—Adenocarcinoma of glandular stomach. Gross specimen. Bar = 10 $\mu$m.

Fig. 3.—Adenocarcinoma of glandular stomach. The neoplastic epithelial cells which retained the characteristic glandular arrangements invaded the stalk. H. & E. Bar = 10 $\mu$m.

Fig. 2.—Polypoid adenoma of glandular stomach. The lesion consists of disorderly elongated and branched glands lined by mucinous epithelium. The stalk is evident. H. & E. Bar = 10 $\mu$m.

Fig. 4.—Adenocarcinoma of glandular stomach. The glandular and tubular elements of malignant gastric mucosa invades the entire wall. The tumour also grows into the lumen. H. &. E. Bar = 10 $\mu$m.

12 animals developed 13 adenocarcinomas. In the treated males, 16 (32%) animals developed 17 tumours of this organ. Of thee, 9 mice had 9 adenomas, 6 mice had 6 adenocarcinomas and 1 mouse developed an adenoma and an adenocarcinoma.

Grossly, the tumours mainly grew into the lumen of the stomach, had irregular shape and were often nodular, ranging from 1 to 12 mm in diameter (Fig. 1).

In the typical benign hyperplasia, the gastric glands exhibited irregular sizes and shape, and were often dilated. The proliferation of mucus epithelium was distinct. The polypoid adenomas usually displayed mushroom-like growths, which consisted of a fibrous central stalk covered by glandular elements of various sizes and shapes, and were lined by tall mucinous epithelium with dark-staining nuclei (Fig. 2). Some adenocarcinomas probably started to grow as polypoid adenomas, but subsequently the neoplastic glands obliterated the central stalk (Fig. 3) and invaded the muscularis layer. On other occasions, the lesions possibly developed into malignancy without the transitory benign stage. In certain instances, the adenocarcinomas extended through the entire wall of the stomach (Fig. 4). The adenocarcinomas were composed of either nests of small acini or elongated glands, and the lining mucinous cells exhibited various stages of differentiation (Figs 5 & 6).

FIG. 5.—Adenocarcinoma of glandular stomach. The large tumour growth obliterates the lumen of stomach. H. & E. Bar = 10 μm.

FIG. 6.—Adenocarcinoma of glandular stomach. The neoplastic gastric glands are lined by cuboidal or columnar cells. The mucin is present in varying amounts in the glands. H. & E. Bar = 10 μm.

Grossly and histologically these tumours were similar to those decribed by other investigators (Sugimura & Fujimura, 1967; Sugimura *et al.*, 1969; Saito *et al.*, 1970).

*Tumours in the solvent-instilled control mice*

The tumour incidences of the glandular stomach in the solvent-instilled control mice were 2% in the females and 0% in the males.

*Tumours in untreated control mice*

No tumours of the glandular stomach were found in either sex of untreated mice. The 3 most common tumours were of the lungs, malignant lymphomas and blood vessels and they occurred in 29, 15 and 7% in females and 19, 10 and 5% in the males, respectively.

The tumour incidences and the survival rates of the solvent instilled and untreated mice will be described in detail in a forthcoming publication.

### DISCUSSION

The findings show that a single intragastric instillation of 4-(hydroxymethyl)-benzenediazonium tetrafluoroborate, given at 400 μg/g body wt, induced tumours in the glandular stomach of Swiss mice. The tumour incidences in the treated animals were 30% ($P < 0{\cdot}00045$) in females and 32% ($P < 0{\cdot}00007$) in males, respectively.

FIG. 7.—Chemical structures of agaritine and 4-(hydroxymethyl)benzenediazonium ion.

Statistical analysis was carried out by the use of Fisher's exact probability test for 2 x 2 tables (Armitage, 1971). Light-microscopic examination of the tumours revealed the characteristic appearances of polypoid adenomas and adenocarcinomas.

The occurrence of 4-(hydroxymethyl)-benzenediazonium ion in *Agaricus bisporus* was demonstrated earlier (Levenberg, 1962). This diazonium ion was also shown by us to be present in the acidic extract of the fungus at a level of 0·6 pts/$10^6$ (Ross *et al.*, 1981, 1982). In addition, it was revealed recently that this ion is generated from the non-carcinogenic agaritine (Toth *et al.*, 1981) by an enzyme system acting *in vitro* in the mushroom (Fig. 7). Interestingly, the activity of this enzyme is only partially lost when exposed to heat used in average cooking (Ross, *et al.*, 1981). Furthermore, our chemical-stability investigation demonstrated that, after moderate cooking (a gentle sauté), mushrooms lost only 47% of their agaritine content (Ross *et al.*, unpublished). Also, when given to mice by gavage, agaritine, a non-carcinogenic hydrazine analogue (Toth *et al.*, 1981) was detected in all digestive-tract segments (Ross *et al.*, unpublished). In view of the above-mentioned findings the research in this field should be intensified, despite the fact that the 4-(hydroxymethyl benzenediazonium ion is unstable. Agaritine, a stable and water-soluble compound when absorbed from the gastrointestinal system, is probably distributed throughout the body, and the likelihood exists that in the various cells the different oxidative enzymes momentarily transform it to the diazonium ion. Since the diazonium compound induced tumours at the application site (*i.e.*, subcutis and glandular stomach) the implications of the findings are self-evident.

In parallel investigations, the 4-(hydroxymethyl)benzenediazonium tetrafluoroborate was mutagenic in *S. typhimurium* TA1535 and TA1537 strains. In addiltion, the carcinogenic acetylated form of 4-hydroxymethylphenylhydrazine was mutagenic in TA1537, without metabolic activation, and it also had marginal DNA-modifying activity with activation. Finally, agaritine gave equivocal results in both *in vitro* assays, showing a small enhancement in mutagenicity in *S. typhimurium* TA1537 without metabolic activation, while having marginal DNA-modifying activity in the presence of S-9 (Rogan *et al.*, 1982).

The worldwide consumption rate of the cultivated mushroom *Agaricus bisporus* is enormous and the demand is increasing (Miller, 1972; Maw & Flegg, 1974). In addition to the diazonium salt, an earlier study demonstrated that the *N'*-acetyl derivative of 4-hydroxymethylphenylhydrazine, another component of this fungus, is carcinogenic in the lungs and blood vessels of mice (Toth *et al.*, 1978). Although both compounds are unstable, the research in this field, particularly in biochemistry, should intensify in case of possible human health hazard implications.

This study was supported by U.S.P.H.S. contract N01-CP05629 from the National Toxicology Program, N.I.E.H.S.

### REFERENCES

ARMITAGE, P. (1971) *Statistical Methods in Medical Research*. Oxford: Blackwell Sci. Publs. p. 136.

DANIELS, E. G., KELLY, R. B. & HINMAN, J. W. (1961) Agaritine: an improved isolation procedure and confirmation of structure by synthesis. *J. Am. Chem. Soc.*, **83**, 3333.

GIGLIOTTI, H. (1963) Studies on the $\gamma$-glutamyltransferase and arylhydrazine oxidase activities of *Agaricus bisporus. Ph.D Dissertation*, University of Michigan.

GIGLIOTTI, H. J. & LEVENBERG, B. (1964) Enzymatic transfer of the $\gamma$-glutamyl group between naturally occurring aniline and phenylhydrazine derivatives in the genus *Agaricus. Biochem. Biophys. Acta*, **81**, 618.

LEVENBERG, B. (1962) An aromatic diazonium compound in the mushroom *Agaricus bisporus. Biochim. Biophys. Acta*, **63**, 212.

LEVENBERG, B. (1964) Isolation and structure of agaritine, a $\gamma$-glutamyl-substituted arylhydrazine derivative from Agaricaceae. *J. Biol. Chem.*, **239**, 2267.

MAW, G. A. & FLEGG, P. B. (1974) The mushroom as a source of dietary protein. In *Glasshouse Crop Annual Report*. Littlehampton: p. 137.

MILLER, O. K. (1972) *Mushrooms of North America*. New York: Dutton & Co. p. 126.

ROGAN, E., WALKER, B., GINGELL, R., NAGEL, D. & TOTH, B. (1982) Microbial mutagenicity and DNA-modifying activity of selected hydrazines. *Mutat. Res.* (In press).

ROSS, A., NAGEL, D. & JAE, H. S. (1981) The identification of aromatic diazonium ions in mushroom extracts. *Proc. Am. Assoc. Cancer Res.*, **22**, 140.

ROSS, A. E., NAGEL, D. L. & TOTH, B. (1982) Evidence for the occurrence and formation of diazonium ions in the *Agaricus bisporus* mushroom and its extracts. *J. Agric. Food Chem.* **30**, 521.

SAITO, T., INOKUCHI, K., TAKAYAMA, S. & SUGIMURA, T. (1970) Sequential morphological changes in *N*-methyl-*N'*-nitro-*N*-nitrosoguanidine carcinogenesis in the glandular stomach of rats. *J. Natl Cancer Inst.*, **44**, 769.

SUGIMURA, T. & FUJIMURA, S. (1967) Tumor production in glandular stomach of rats by *N*-methyl-*N'*-nitro-*N*-nitrosoguanidine. *Nature*, **216**, 943.

SUGIMURA, T., FUJIMURA, S., KOGURE, K. & 6 others (1969) Production of adenocarcinomas in the glandular stomach of experimental animals by *N*-methyl-*N'*-nitro-*n*-nitrosoguanidine. *Gann Monogr.* **8**, 157.

TOTH, B. (1972) A toxicity method with calcium cyclamate for chronic carcinogenesis experiments. *Tumori*, **58**, 137.

TOTH, B., NAGEL, D., PATIL, K., ERICKSON, J. & ANTONSON, K. (1978) Tumor induction with the N'-acetyl derivative of 4-hydroxymethylphenylhydrazine a metabolite of agaritine of *Agaricus bisporus. Cancer Res.*, **38**, 177.

TOTH, B., NAGEL, D. & ROSS, A. (1980) Occurrence and the carcinogenic action of 4-(hydroxymethyl)-benzenediazonium ion (4-HMBD). *Proc. Am. Assoc. Cancer Res.*, **21**, 73.

TOTH, B., PATIL, K. & JAE, H. S. (1981) Carcinogenesis of 4-(hydroxymethyl(benzenediazonium ion (tetrafluoroborate) of *Agaricus bisporus. Cancer Res.*, **41**, 2444.

TOTH, B., RAHA, C. R., WALLCAVE, L. & NAGEL, D. (1981) Attempted tumor induction with agaritine in mice. *Anticancer Res.*, **1**, 255.